# The influence of cost-per-DALY information in health prioritisation and desirable features for a registry: a survey of health policy experts in Vietnam, India and Bangladesh

**DOI:** 10.1186/s12961-016-0156-6

**Published:** 2016-12-03

**Authors:** Yot Teerawattananon, Sripen Tantivess, Inthira Yamabhai, Nattha Tritasavit, Damian G. Walker, Joshua T. Cohen, Peter J. Neumann

**Affiliations:** 1 Health Intervention and Technology Assessment Program, Ministry of Public Health, Nonthaburi, Thailand; 2 Bill and Melinda Gates Foundation, Seattle, WA USA; 3 Tufts Medical Center, Tufts University, Boston, MA USA

**Keywords:** Cost per DALY, Economic evaluation, Registry, Database, Policy decisions, Low- and middle-income countries

## Abstract

**Background:**

Economic evaluation has been implemented to inform policy in many areas, including coverage decisions, technology pricing, and the development of clinical practice guidelines. However, there are barriers to evidence-based policy in low- and middle-income countries (LMICs) that include limited stakeholder awareness, resources and data availability, as well as the lack of capacity to conduct country-specific economic evaluations. This study aims to survey health policy experts’ opinions on barriers to use of cost-effectiveness data in these settings and to obtain their advice on how to make a new cost-per-DALY database being developed by Tufts Medical Center more relevant to LMICs. It also identifies the factors influencing transferability.

**Methods:**

In-depth interviews were conducted with 32 participants, including policymakers, technical advisors, and researchers in Health Ministries, universities and non-governmental organisations in Bangladesh, India (New Delhi, Tamil Nadu and Karnataka) and Vietnam.

**Results:**

The survey revealed that, in all settings, the use of cost-effectiveness information in policy development is lacking, owing to limited knowledge among policymakers and inadequate human resources with health economics expertise in the government sector. Furthermore, researchers in universities do not have close connections with health authorities. In India and Vietnam, the demand for evidence to inform coverage decisions tends to increase as the countries are moving towards universal health coverage. The informants in all countries argue that cost-effectiveness data are useful for decision-makers; however, most of them do not perform data searches by themselves but rely on the information provided by the technical advisor counterparts. Most interviewees were familiar with using evidence from other countries and were also aware of the influences of contextual elements as a limitation of transferability. Finally, strategies to promote the newly developed database include training on basic economic evaluation for policymakers and researchers, and effective communication programs, with support from reputable global agencies.

**Conclusions:**

Although cost-effectiveness information is recognised as essential in resource allocation, there are several impediments in the generation and use of such evidence to inform priority setting in LMICs. As such, the Cost-per-DALY database should be well-designed and introduced with appropriate promotion strategies so that it will be helpful in real-world policymaking.

**Electronic supplementary material:**

The online version of this article (doi:10.1186/s12961-016-0156-6) contains supplementary material, which is available to authorized users.

## Background

Countries increasingly recognise the need to allocate limited healthcare resources efficiently [1]. To this end, economic evaluation has been implemented in many areas, including coverage decisions, technology pricing and the development of clinical practice guidelines [2, 3]. Although economic evaluation is much more prevalent in high-income countries (HICs) [4], it may in principle make a bigger impact in low- and middle-income countries (LMICs) given the pressing needs in those settings [5, 6]. However, there are barriers to evidence-based policy in these countries that include limited stakeholder awareness, resources and data availability [7, 8], as well as the lack of capacity to conduct country-specific economic evaluations [9]. Given the wide practice of using economic evidence to inform resource allocation in HICs, there are a number of studies that address these issues in these settings [10–12]. On the contrary, literature in LMICs is lacking despite the significant need.

The development of a database that systematically catalogues the literature would benefit decision-makers in LMICs, which lack capacity in health economics [13, 14]. Existing examples include the database at the University of York’s Centre for Reviews and Dissemination and Tufts Medical Center’s Center for the Evaluation of Value and Risk in Health (CEVR) Cost-Effectiveness Analysis (CEA) Registry (www.cearegistry.org). Most of the studies included in these databases are from HICs because the numbers of economic evaluations, especially cost-per-Quality Adjusted Life Year (QALY) analysis, in HICs are much higher than the LMICs counterpart. The strength of the University of York database is its inclusion of studies using either of two health metrics (QALY and Disability Adjusted Life Year (DALY)), and its provision of quality appraisals for each study. However, this database does not synthesise results from multiple studies in a single table, and its query function is limited (for example, it is not possible to search based on target population age or study perspective) [15]. Furthermore, the York Centre for Reviews and Dissemination database has discontinued updates. On the other hand, the Tufts CEA Registry synthesises results, but includes only cost-per-QALY studies and is not completely publicly accessible [16].

The relative merits of the cost-per-DALY averted and cost-per-QALY measures have been debated extensively [17–19]. Although the literature contains far more cost-per-QALY articles than cost-per-DALY articles, the latter metric is more common in studies of LMICs because the international development organisations that fund this work, such as the WHO and the Bill & Melinda Gates Foundation (BMGF), favour use of DALYs [20]. The WHO Cost-Effectiveness and Strategic Planning (WHO-CHOICE) program has a compilation of cost-per-DALY studies, but its contents are limited to WHO-sponsored studies [21].

Recognising the importance of a cost-per-DALY database for LMICs, the BMGF has funded Tufts Medical Center’s CEVR to develop a cost-per-DALY Registry. The Registry aims to inform decision-makers on how to best improve health resource allocation and to enhance the use of standard methodologies by technical advisers. However, because economic evaluations are not necessarily generalisable^1^ or easily transferable to local settings,^2^ development of a database per se does not ensure its use. This is the first qualitative study to determine the barriers that influence use of cost-effectiveness data in LMICs and obtain advice from relevant stakeholders on how to make the new cost-per-DALY database being developed by CEVR more relevant to these settings. It also explores the usefulness of a cost-per DALY database to health policymakers and other stakeholders, and identifies the factors influencing transferability. Given that cost-per-DALY analysis has been primarily conducted in LMICs, the majority of studies included in the new Registry will be from resource-poor settings, dissimilar to cost-per-QALY databases that focus on HIC studies.

## Methods

### Samples: settings

The study settings were purposively chosen. The selected countries – Bangladesh, India and Vietnam – though limited to Asia, represent a range of health expenditures per capita and coverage of public health insurance [22]. Because India’s health systems are run by its constituent states, three states were selected to represent central and state health systems (including one richer and one poorer state) and to represent a range of development. Interviews were conducted in Dhaka, Bangladesh; New Delhi (where the federal government is located); Bangalore and Karnataka (low income and less developed health systems); Chennai, Tamil Nadu (higher income and more developed health systems); and Hanoi, Vietnam. Other factors considered in the selection of study settings were a lack of established health technology assessment (HTA) systems, limited economic evaluation capacity and the feasibility of traveling from Thailand to conduct face-to-face interviews.

Table 1 describes the study settings. The health expenditure per capita in Bangladesh, India and Vietnam is US$67.8, US$157 and US$234, respectively [23]. Meanwhile, the coverage of government health schemes varies from 1% in Bangladesh to 20% in India and 60% in Vietnam. The proportion of healthcare that is paid for out-of-pocket is relatively high in these three countries.

**Table 1:** Background information of Vietnam, India and Bangladesh

Country/States	Bangladesh	India	Tamil Nadu	Karnataka	Delhi	Vietnam
Population^a^ (million)	155	1240	72	64	9	91
GDP per capita^b^ (Current US$, 2012)	750	1503	1844	1441	3762	1755
Health expenditure per capita^a^ (% of GDP)	67.8 (6.6%)	157 (4.1%)	N/A	N/A	N/A	234 (6%)
Out-of-pocket as a percentage of total expenditure on health^a^	63%	58%	N/A	N/A	N/A	49%
Percentage of people covered by public health insurance	1% (24)	20% (25)	18% (26)	N/A	N/A	60% (27)
Year by which they plan to achieve Universal Health Coverage	2032	2022				2020

Sources: ^a^WHO [23]; ^b^World Bank [28], Planning Commission, Government of India [29]

### Document review

Reviews of relevant documents were conducted during December 2013 to February 2014 as a means to gather information on the health systems context and existing capacity for economic evaluation in the study settings. The reviews involved reports issued by country/state governments and international agencies, as well as published articles in peer-reviewed journals. Together with interview information, these documents help enhance understanding on the roles of health authorities, current direction of and progress in health sector reforms, need for evidence to guide resource allocation, and limitations in health economic capacity.

### In-depth interviews

Semi-structured, in-depth interviews with key stakeholders were employed as the major approach for data collection in this study.

### Samples: interviewees

The interviewees were initially identified from networks of the Health Intervention and Technology Assessment Program (HITAP), namely the CAPacity building program on Universal Health Coverage and the Mahidol University Global Health program in Thailand, and the National Institute for Health and Care Excellence’s International Unit, United Kingdom. The search targeted decision-makers responsible for health resource allocation at the national- or state-level; technical officers, including researchers and those who advise health policymakers; or policy analysts in non-profit organisations with good knowledge of the local context.

Invitations to participate were sent via emails to describe the study’s purpose and the interview protocol. A snowball technique was also employed to identify relevant interviewees (about 2–3 persons). Fifty-one participants were invited, including 12 from Bangladesh, 28 from the three Indian states, and 11 from Vietnam. The response rate was 63% (32 participants), including 10 from Bangladesh, 15 from India, and 7 from Vietnam. Profiles of the interviewees are shown in Table 2.

**Table 2:** Profiles of the interviewees

Country	Vietnam	India (Tamil Nadu, Karnataka, and New Delhi)	Bangladesh
Number of policymakers at Ministries of Health including senior officials, such as Director of departments, but excluding politicians	3	5	4
Number of technical advisors within Ministries of Health and academic units related to health economics, health policy, and health financing	4	10	4
Number of staff of NGO in development and health sectors	–	–	2
Total	7	15	10

### Interviews

During January to March 2014 four HITAP staff members conducted the interviews, using interview materials. Samples of the results by search options are shown in the supporting information section. Basic search is used for quick search for articles, ratios and utility weights. Briefly, results by articles show detailed information (i.e. publication year, journal and article title) of retrieved papers matched with the search term. Results by ratio show cost-per-DALY information of each paired intervention and its comparator in a given setting of selected studies. Results by weight show disability weight of each health condition in selected studies. Since the background of interviewees are very varied and some might not have used economic evaluation results before, the researchers provided brief information of the concept of DALYs and a real case study of an economic evaluation conducted in Thailand in order to facilitate the discussion.

Advanced search can be used to search for more complex queries and specific issues. Users can choose to select country of interest, publication date, study themes, intervention type, prevention stage, disease, time horizon, perspective, or quality of study. Moreover, they can search by specific ratio, impact type and budget impact, and utility weights information.

IY and NT were involved in all settings, and YT and ST alternated between study settings. This scheme ensured the interviews were administered consistently across all settings. Semi-structured interviews were conducted in English with decision-makers and technical officers to gather their views on: (1) the current situation of health decision-making and the role of economic evaluation as well as other types of evidence; (2) the usefulness of cost-per-DALY information; (3) desirable characteristics of a cost-per DALY database; and (4) factors influencing the transferability of cost-per-DALY information across countries. The interview guide questions are listed in the supporting information section. An interpreter was provided in the case of some of the respondents from Vietnam. Each interview lasted 40 to 50 minutes. Interview sessions were tape-recorded unless otherwise requested. In some cases and as requested, the interview materials were sent to the interviewees after the interview for further comments and suggestions.

### Data analysis

Interviews were transcribed non-verbatim for qualitative thematic analysis. Key elements (themes and subthemes) were identified from transcripts, as informed by the study objectives, related interview questions and other factors. These factors included the interviewee’s understanding of economic evaluation and the cost-per-DALY metric; processes and information requirements for health policy decisions; economic evaluation capacity of the country (including capacity in the Ministry of Health (MoH), universities and research institutes); the interviewee’s perception of the usefulness of cost-per-DALY information; his/her opinion about desirable database characteristics and the transferability of cost-effectiveness evidence; and interviewee suggestions to enhance the database and general comments. Thematic statements were arranged under relevant headings. IY, NT and ST independently reviewed and interpreted the retrieved information. A meeting was organised for the three researchers to exchange their findings. For the issues that generated different opinions, corresponding interview transcripts were revisited and the discussion was carried on until consensus was reached.

## Results

### Economic evaluation in Bangladesh

In 2012, Bangladesh adopted a universal health coverage (UHC) program guided by a health financing study; however, current insurance coverage is less than 1% of its total population [24]. The Ministry of Health and Family Welfare (MoHFW) plays a pivotal role in health policies formulation. External sources of finance account for 7.2% of total health expenditure [25], with major donors including the World Bank and the Asian Development Bank [26]. Resources are allocated through the Health Population Nutrition Sector Development Program 2011–2016. The 32 operational plans of this program highlight the competing demands for limited resources. In addition, BRAC (an NGO), substantially contributes to the funding, development and implementation of public health programs in this country.

Economic evaluation expertise in Bangladesh is limited, although there are two health economics units in the MoHFW, University of Dhaka and International Center for Diarrheal Disease Research, Bangladesh (icddr,b) – a non-governmental research institute. The interviewees explained that the number of health economists is inadequate. While both the demand for health economists in the MoHFW and the number of trained students has been growing, government officer positions have not likewise increased. As a result, graduates from the University do not work in health economics. In addition, local data are not available and the budget for data collection is insufficient. This results in the limited number of economic evaluation studies available locally. Most health economics studies focus on costs only. The icddr,b conducts cost-effectiveness analysis of some public health interventions developed by its researchers, and also offers short-course trainings on health economics to health personnel. Nevertheless, such an effort to build capacity in this area yields limited impact in both academic and policymaking spheres.

### Economic evaluation in India

In India, the federal government is responsible for national strategic planning (e.g. the National Rural Health Mission and Urban Health Initiative), for health regulation (e.g. professional practice, health product regulation, etc.), international health, and subsidisation for poor states. The federal government planned to achieve UHC by 2022, which will require a substantial increase in health expenditure [27], while the current health spending is one of the lowest in the region and indicates that resources might prove a constraint in scaling up, necessitating better tools for prioritisation. However, the capacity of the MoH and Ministry of Finance (MoF) on the costing of public health policies is limited and should be strengthened before introducing economic evaluation.

According to the Indian Constitution, each state has its own healthcare system and is responsible for the health of its respective population. The selected states provide public health insurance for poor families up to a certain level. For example, in Karnataka, the Vajpayee Arogyashree scheme, introduced in 2010 for those below the poverty line, focuses only on tertiary healthcare in seven specialties (cardiovascular, oncology, renal diseases, neurosurgery, neonatal surgeries, burns, and polytrauma) [28]. There are other similar state governments’ schemes running in a number of states.

Interviewees said that the use of economic evaluation to inform policy in India is currently in its initial stages and there is not enough understanding of it. The dual source of policymaking – at the Centre and at the states – makes the situation more complex. Similar to the federal government, economic evaluation is not used by the state governments because decision-makers currently do not demand it or lack the capacity to supply economic evidence. Moreover, there is no official mechanism to include cost-effectiveness evidence in the decision-making process. Nevertheless, the research capacity of economic evaluations is available in academic institutes, some of which are well recognised at the global level.

### Economic evaluation in Vietnam

Health resource allocation in Vietnam is mainly undertaken by multi-departmental committees, chaired by the Health Minister. Requirements for formulation of the medicine reimbursement list include clinical efficacy, market approval, prescriber experience and prices. According to interviewees, due to severe fiscal constraints in recent years, decision-makers now pay significant attention to budget implications and value for money when evaluating new technologies and interventions.

During the last 5 years, the MoH implemented a policy to build the country’s HTA capacity to achieve sustainable UHC by 2020 [27]. This initiative will increase the demand for cost-effectiveness evidence. On the supply side, the Health Strategy and Policy Institute, which resides within the MoH, is responsible for generating evidence.

There is an increasing interest to incorporate economic evaluation in coverage decisions under the UHC program managed by the Vietnamese Social Security. The Health Strategy and Policy Institute has been tasked to review the existing pharmaceutical and health services packages with the aim to introduce the new Basic Health Service Package by 2018. A systematic review of economic evaluation conducted in Vietnam reveals that, in order to meet the government policy demand, there is an urgent need to improve technical capacity of local research scholars/institutes, and in some instances, the transfer of health economic evidence from countries in the same region will be helpful for policymaking [28].

### Usefulness of cost-per-DALY information

A greater role for economic evaluation in these settings can be anticipated given the pledge by Vietnam and India to introduce UHC. There is increasing pressure to adopt new and high-cost technologies, and most of the interviewees in these settings agree that cost-per-DALY information is critical. However, they also noted that policymakers rarely search for this kind of information. Their decisions are instead based on data analysis results and other information provided by the technical advisor counterparts who serve as the committees’ secretariat.

The interviews revealed that the cost-per-DALY concept is not well understood among most policymakers and technical officers. It is also not well-understood among many researchers and physicians who sit on the decision-making committees. This was evident in Vietnam, even though this country has used ad hoc cost-effectiveness information to formulate health-related policies such as coverage for some expensive medicines. The gap in knowledge can be attributed to the lack of institutionalisation of health economics in the MoH. Instead, in the countries studied, the focus is on cost and budget implications. Many interviewees recognised that this focus biases policy towards low-cost interventions while sometimes rejecting interventions with better outcomes but higher costs. For this reason, these interviewees stated that a well-designed cost-per-DALY database would be very useful.

### Desirable characteristics of a cost-effectiveness database

Most interviewees argued that the Tufts CEA Registry, as described by the researchers and illustrated in the interview materials, was not user-friendly. A major complaint was that the existing Registry does not allow users full access to the database via advanced search (available in ‘premium access’) if they are not a paid member. In addition, the database does not allow users to download or extract results to other formats such as excel file. They added that the database should be freely accessible, be available online, require no special software to use, and should include an online user manual. Further suggestions are provided below.

### Search options

Two of the interviewees recommended use of World Bank geographic classifications (e.g. Asia, Africa and Latin America) and economic development levels (i.e. low, lower-middle, upper-middle and high income). Furthermore, they recommended having provincial-level information for larger countries such as India and China. It was also suggested that the search options are standardised globally and searchable using the International Classification of Diseases system. For demographic groups, many interviewees suggested use of broadly recognised categories, e.g. children under 5 years old, teenagers and women of reproductive age, because these terms are in line with the design of public health programs. Most interviewees in Bangladesh and India suggested the inclusion of vulnerable groups or poor people since they are of high concern to the governments in these countries.

Finally, it was recommended that the database be searchable using multiple keywords combined using Boolean operators (AND, OR, NOT). If appropriate, drop down options or autocomplete function should be used. Definitions should be provided for terms not familiar to persons outside particular disciplines. For example, the term ‘time horizon’ may not be well understood by users who are not health economists.

### Contents

The cost-per-DALY database should contain reliable, updated and comprehensive data. Technical officers recommended inclusion of reputable “grey” literature such as reports produced by reliable sources like the WHO, BMGF and national governments. All economic evaluation studies in this database should be graded for their technical robustness using international classification standards. In addition, the database should be able to characterise changes in cost-effectiveness information for particular interventions over time and across countries or regions. The interviewees recommended inclusion of both cost-per-DALY and cost-per-QALY estimates in a single consolidated database for LMICs.

Furthermore, most technical officers and researchers interviewed in this study recommended reporting the perspective used by each study, the study sample size and budget impact estimates, if available. Ex-ante model-based economic evaluation (on unimplemented policies) versus ex-post economic evaluation (using data from real policy implementation) were also identified to be coded differently. The interviewees explained that they are likely to value ex-post evaluations more than ex-ante ones since the former is perceived to be proven as opposed to something assumed.

### Results presentation

In the interviews, health economists and technical officers cited the CEA Registry’s synthesis of results from multiple studies in a single table as helpful. They also suggested that the search results be presented in various formats, such as graphs, diagrams or figures so that they are easy to understand. A few interviewees referred to the Institute of Health Metrics and Evaluation’s data visualisation [29]. Some interviewees requested the display of additional information, including the study setting context, e.g. whether UHC has been implemented, how much of the country’s output is spent on health, who pays for interventions and disease burden.

The interviewees recommended use of standard terms, including nominal and international dollars^3^ and provide information for calculation purposes. The database should report on potential conflicts of interests, i.e. funding. Subjective quality scores^4^ should be accompanied by a description of the methods used. Ethical issues and disclaimers should also be clarified. The interviewees stated incorporation of search screens and output formats similar to those used by the WHO or the World Bank would facilitate use of the cost-per-DALY database.

### Transferability

Most interviewees were familiar with the influence of contextual factors on the use of overseas data in their country. Although the most senior decision-makers in Vietnam, such as the Health Minister and Vice Ministers, requested technical staff to include a chapter on international literature reviews in policy proposal documents, information from countries such as Europe and North America are only used as reference or background information. Conversely, information from surrounding geographic areas with similar cultures or health systems (such as Thailand in the case of India and Vietnam or India and Nepal in the case of Bangladesh), are most influential.

Ten health economist interviewees pointed out that given the differences in the health systems, researchers should not rely on economic evaluations conducted in other countries. The main barriers to using cost-effectiveness information across countries are differences in costs and clinical practice. No interviewee accepted the generalisability of cost-effectiveness information across settings, but they did agree that economic evaluation studies can be used in different settings if locally relevant information is used to develop model assumptions. As a result, studies in the database can be useful for providing background information, analytical frameworks, retrieving clinical efficacy or effectiveness data, and comparing results.

Interestingly, the interviewees implied that the interpretation of the information provided by the database should not be based on the conclusion of the majority of studies, but rather on the conclusions of studies that are most contextually relevant. For example, if most studies for a particular intervention indicate it is cost-effective but the study settings are not representative of the user’s setting, the results will be less meaningful than results from a more limited number of studies that are representative of similar settings.

### Promoting the database in LMICs

Economic evaluation should be promoted among key figures in the MoH and MoF. Other targeted users should include policymaking committees, leading health professionals and academicians, and non-health sector decision-makers.

As suggested by the respondents, a database should be introduced along with efforts to improve knowledge about cost-effectiveness analysis and the capacity to conduct it. The joint introduction of the database and the education efforts will help ensure that the database is not used inappropriately. It will also give users an opportunity to learn how to present the data extracted from the database so that it is relevant to decision-makers. Training workshops should be held to provide hands-on experience and promote an understanding of the cost-per-DALY metric. The database can be integrated as part of the strategy of moving towards UHC. Workshops can be held through a regional or global initiative such as the WHO regional workshops on UHC or Joint Learning Network on UHC (www.jointlearningnetwork.org).

Additionally, a communication strategy should be planned. The CEVR team should collaborate with reputable organisations such as the WHO or World Bank to help promote the database to its Member states and partners. The CEVR might also consider coordinating with HTA networks such as the International Society for Pharmacoeconomics and Outcomes Research (ISPOR), Health Technology Assessment international (HTAi), and the International Decision Support Initiative (iDSI). Social networks can be an effective channel to inform individuals.

### Monitoring and evaluation

The database should be monitored and evaluated, especially during the first 1–2 years following its introduction, with feedback used to guide further development. The interviewees in Bangladesh suggested that the database website include a feature to allow users to communicate with the developers, e.g. via blogs, comment boxes or electronic feedback forms. This feature can serve many purposes, including identification of studies that should be included in the database or notification of incorrect database information.

Some interviewees also mentioned that external assessment could be conducted by a third party on an ad-hoc basis to create trust among users regarding the database’s quality and reliability. Building this kind of trust is crucial to promoting the database’s legitimacy among potential government users.

## Discussion

Respondents in this study confirmed that the cost-per-DALY registry would be useful for LMICs, which typically have limited economic evaluation capacity. Characteristics that would encourage database use include:

1. Rather than cost-effectiveness league tables that rank health investment independent of disease areas and target population groups, decision-makers often want information to evaluate investments to address particular problems for particular groups, e.g. whether and how to control tuberculosis among migrant workers. Search functionality should ensure that the database can address a wide range of specific policy questions.
2. Although this study focused on the cost-per-DALY metric, the database should also include cost-per-QALY information from the same search engine. This is because the users want to make the best use of all available information rather than selective information when making policy decisions.
3. If the registry targets national governments, it must establish credibility by gaining endorsements by reputable global health authorities. Interviewees suggested strategic collaborations with major global health partners and rigorous internal and external monitoring. External monitoring should be conducted by an independent body, preferably a consortium of partners, including experts from international governmental organisations, HTA agencies from LMICs, and academics from target countries. Meanwhile, partners should be identified to promote the database in their individual countries and to identify unpublished studies from their countries for inclusion in the database.
4. The database should improve local capacity for health economics by including basic information on research methods, and by promoting the conduct of future studies for specific settings. For example, the database website describes the limitations of published studies and how the same intervention can produce different results in different settings. The BMGF should not only fund database promotion but also support training sessions, activities or workshops for countries that are ready to conduct local economic evaluations. Technical officers from the MoH and MoF are among the top priorities.

Given that, when time passes, the price of a technology usually decreases and prescribers acquire experience about the use of particular technologies that result in better health outcomes, the cost-effectiveness results will change in a desirable way. Such an occurrence should be revealed by the database when it includes cost-effectiveness studies of the same health technology that are conducted at different times.

This study has limitations. First, the limited number of interviewees might have an effect on the breadth of the information acquired. However, as we observed during the data collection, there was general agreement among different interviewees on the key issues, suggesting that the weight of this limitation on the key findings may not be significant. Second, although this research focused on informing decision-making at the national level, other groups could also be relevant, such as international donors, who fund vertical programs in LMICs; intergovernmental organisations, who offer policy recommendations to country governments; academics, who may use the database for teaching purposes; and industry, which can use the information for research and development planning or marketing purposes. Third, because our interviews were limited to three countries, generalisations should be made with caution.^5^ Furthermore, the interviewees were identified through international networks of HITAP, which may pose biases, such as obtaining results that are favourable to economic evaluation evidence. Fourth, some of the interviewees had limited knowledge of or experience with economic evaluation. As a result, some may have misinterpreted or misunderstood the issues that the survey explored. Nevertheless, if the interviewers recognised such a problem, they tried to provide explanation and clarification. Finally, the interview materials were taken from the existing Tufts CEA Registry, and not from the cost-per-DALY Registry because the latter was still under development during the data collection phase of this study. In addition, the interview materials were paper-based, making it impractical for interviewees to truly test the website.

The findings from this study should prove helpful for the further development and refinement of the Tufts cost-per-DALY database, which is now available, and for future outreach efforts to engage with decision-makers about resource prioritisation using cost-per-DALY studies. In particular, based on feedback received in this study, as well as other outreach efforts, the team at Tufts Medical Center, which developed the cost-per-DALY database, is considering a series of initiatives, including constructing a list of interventions that represent ‘good value’ in different regions and countries; developing education and training opportunities based on the data; improving the estimation of costs, so that they reflect the needs of local decision-makers, and examining the generalisability of studies conducted in one context to another.

## Conclusions

Although cost-effectiveness information is recognised as essential in resource allocation, especially in resource-finite settings, there are several impediments in the generation and use of such evidence to inform policy in LMICs. As such, the well-designed cost-per-DALY database is considered helpful among technical advisors and health economic researchers. At the same time, respective institutes at national and international level should introduce contextually-appropriate strategies to promote the use of this registry in real-world policymaking.

## Additional files

Additional file 1: Interview material 1: Search results by using articles option. (TIF 404 kb)
Additional file 2: Interview material 2: Search results by using ratio option. (TIF 514 kb)
Additional file 3: Interview material 3: Search results by using weight option. (TIF 718 kb)
Additional file 4: Interview questions. (DOCX 13 kb)

## Abbreviations

BMGF: Bill & Melinda Gates Foundation
CEA: Cost-effectiveness analysis
CEVR: Center for the Evaluation of Value and Risk in Health
DALY: Disability Adjusted Life Year
HICs: High-income countries
HITAP: Health Intervention and Technology Assessment Program
HTA: Health technology assessment
icddr,b: International Center for Diarrheal Disease Research, Bangladesh
LMICs: Low- and middle-income countries
MoF: Ministry of Finance
MoH: Ministry of Health
MoHFW: Ministry of Health and Family Welfare
QALY: Quality Adjusted Life Year
UHC: Universal health coverage

## Footnotes

1 Generalisability is the degree to which the results of a study or systematic review can be extrapolated to other circumstances, in particular to routine healthcare situations.

2 Transferability is the ability to apply information and/or data from one setting to the user’s target setting and is dependent on context specificity.

3 A nominal value is an economic value expressed in historical nominal monetary terms while an international dollar is a value that has been adjusted and measured in terms of the Purchasing Power Parity.

4 A subjective rating score by reviewers from 1 (lowest quality) to 7 (highest quality). Scores reflect the following considerations (in order of importance): (1) whether or not the authors correctly computed the incremental cost-effectiveness ratios; (2) whether or not the authors comprehensively characterised the uncertainty of the results (see sensitivity analysis); (3) whether or not the authors correctly used and explicitly specified the health economic assumptions used in the study (e.g. discount rate, currency, time horizon); and (4) whether or not the authors appropriately and explicitly estimated the utility weights.

5 As part of the same project, we also conducted a parallel study using an electronic survey focusing on the usefulness and transferability of economic evaluation, which covers a range of countries in the Asian, African, and South American regions.
