# Supplementary figures and images for: The influence of cost-per-DALY information in health prioritisation and desirable features for a registry: a survey of health policy experts in Vietnam, India and Bangladesh
Source: Health Res Policy Syst. 2016 Dec 3;14:86. doi: 10.1186/s12961-016-0156-6 (PMC5135838; doi:10.1186/s12961-016-0156-6)

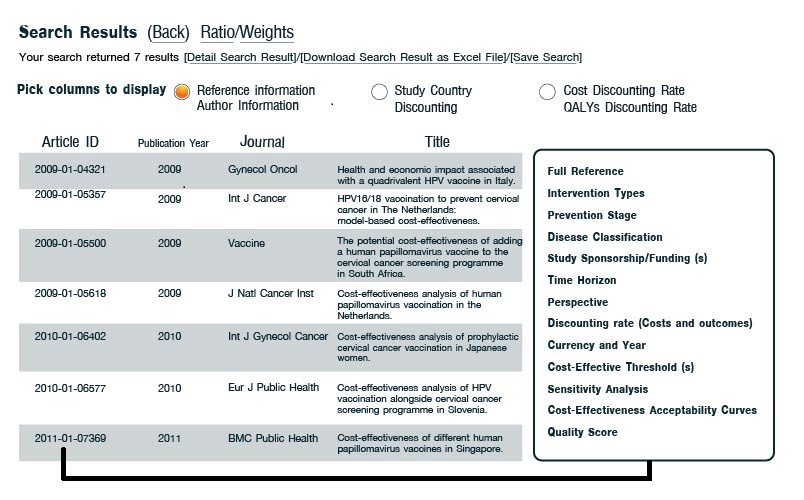

Supplement: Additional file 1: — Interview material 1: Search results by using articles option. (TIF 404 kb) [file 12961_2016_156_MOESM1_ESM.tif]

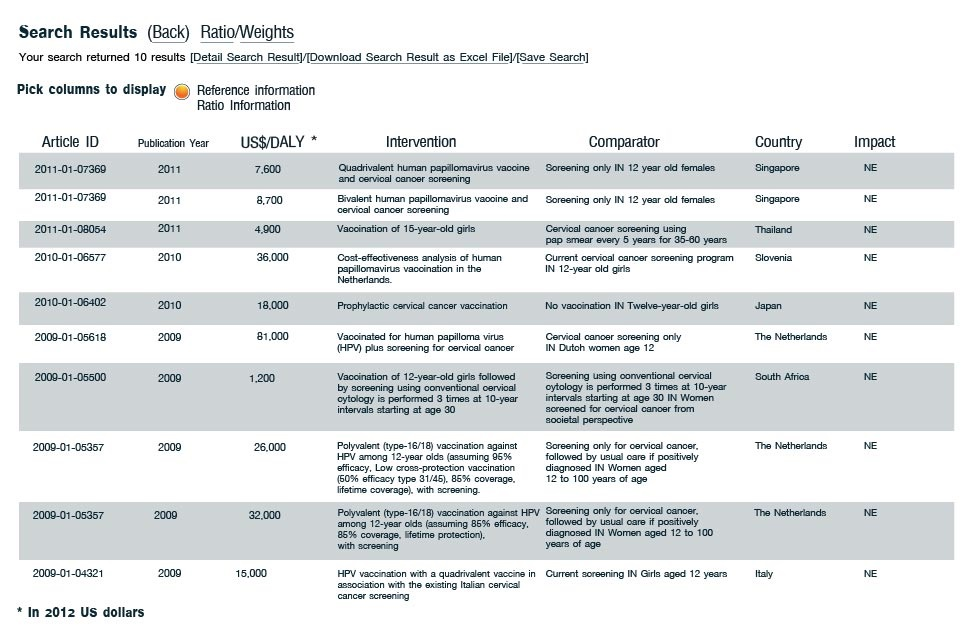

Supplement: Additional file 2: — Interview material 2: Search results by using ratio option. (TIF 514 kb) [file 12961_2016_156_MOESM2_ESM.tif]

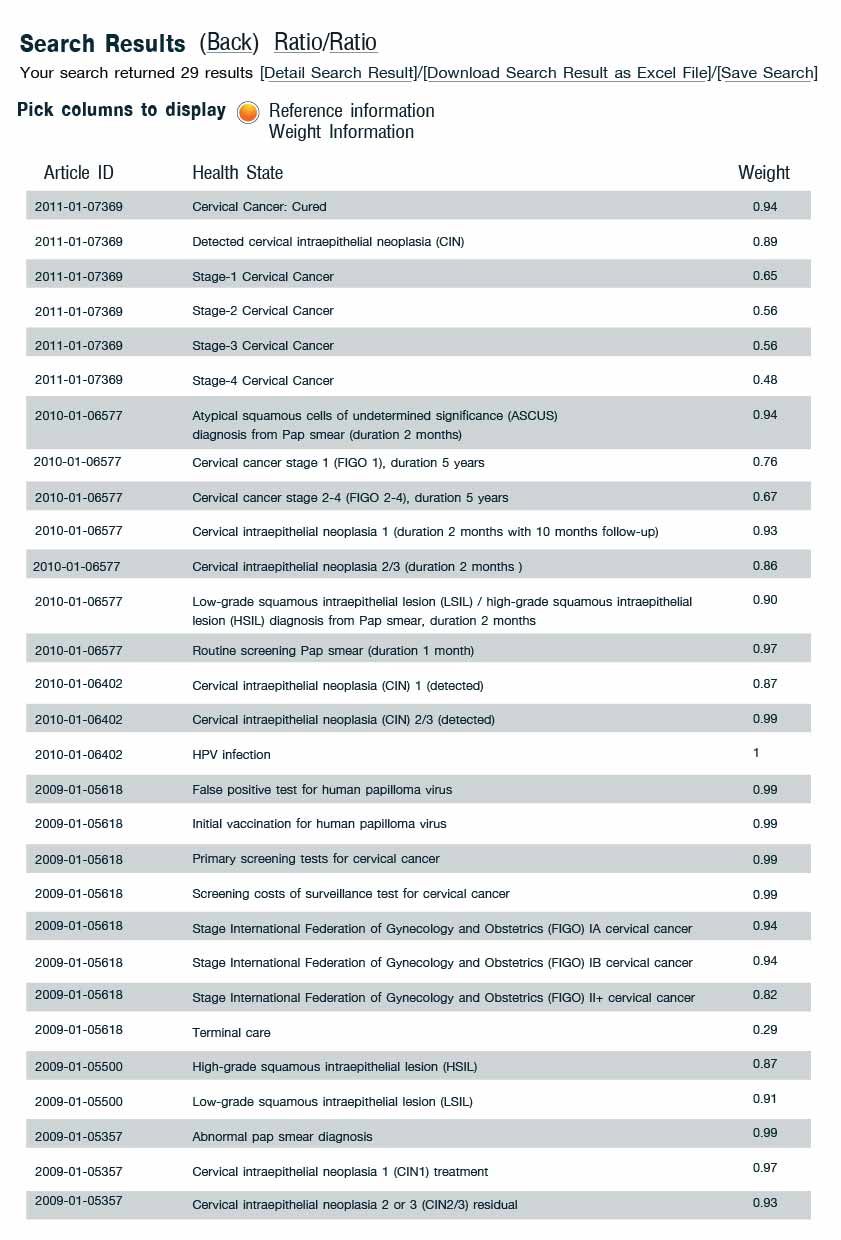

Supplement: Additional file 3: — Interview material 3: Search results by using weight option. (TIF 718 kb) [file 12961_2016_156_MOESM3_ESM.tif]
