# Supplementary material for: The influence of cost-per-DALY information in health prioritisation and desirable features for a registry: a survey of health policy experts in Vietnam, India and Bangladesh
Source: Health Res Policy Syst. 2016 Dec 3;14:86. doi: 10.1186/s12961-016-0156-6 (PMC5135838; doi:10.1186/s12961-016-0156-6)
Supplement: Additional file 4: — Interview questions. (DOCX 13 kb) [file 12961_2016_156_MOESM4_ESM.docx]

**Additional file 4 – Interview questions**

**Interview questions for decision makers**

1. What is your role regarding public health resource allocation? How long have you been in this role?
2. What are the effects of your decisions in question 1 to the public?
3. Regarding resource allocation, what is the governance structure (i.e. committees, mechanisms), criteria, types of information/evidence used for making the decisions?
4. Please give us an example of one of the most difficult decisions you have made. (*N.B.* this question is to triangulate and get more detailed information from questions 1 to 3)
5. Have you ever consulted sources of information/evidence from other countries to inform decision making in your country? If so, please give an example. If not, please tell us why you haven’t used information/evidence from outside your country.
6. (The researchers give examples or case studies of cost-per-DALY studies in order to assess the interviewees’ understanding of state-of-the-art economic evaluations. For those with a low level of understanding, we will provide background information to ensure they are well informed of what we are going to ask in the next step.) Is cost-per-DALY information relevant in informing your decisions?
7. What are the barriers to using cost-per-DALY information, including means of getting information, availability of and capacity to use information, and institutional and social barriers?
8. Apart from cost-per-DALY, what are other types of information necessary for decision making?
9. If global donors such as the BMGF want to invest in programs to help decision makers in low- and middle-income countries make informed decisions, what kind of programs would you like to see?

**Interview questions for technical officers**

1. What is your role in informing resource allocation at the national/state level? How long have you been in this role?
2. Please give us an example of a success story of informing decision makers and key success factors.
3. Please give us an example of decisions that deviated from your advice, why it happened, and what you would do to avoid the same situation.
4. Have you ever used global databases in your work? Please give us an example and reasons for using it.
5. Given your experience, in terms of database features i.e. accessibility, user-friendly, up-to-date, search queries, etc., which global database is the best and why?
6. (The researchers give examples or case studies of cost-per-DALY studies in order to assess the interviewees’ understanding of state of the art of economic evaluations. For those with a low level of understanding, we will provide background information to ensure they are well informed of what we are going to ask in the next step.) Is cost-per-DALY information useful in producing advice to inform decision making?
7. Have you ever used cost-per-DALY to inform decision makers? If yes, can you give us an example? If no, can you tell us why?
8. Do you think cost-per-DALY studies in other settings are relevant in informing decisions in your own setting?
9. (The researchers provide examples of limitations of using cost-per-DALY information in other settings) How can you make the best use of information conducted in other settings to inform the decisions in your setting?
